# Effects of metal ions on caspase-1 activation and interleukin-1β release in murine bone marrow-derived macrophages

**DOI:** 10.1371/journal.pone.0199936

**Published:** 2018-08-23

**Authors:** Maxime-Alexandre Ferko, Isabelle Catelas

**Affiliations:** 1 Department of Mechanical Engineering, University of Ottawa, Ottawa, Ontario, Canada; 2 Department of Surgery, University of Ottawa, Ottawa, Ontario, Canada; 3 Department of Biochemistry, Microbiology and Immunology, University of Ottawa, Ottawa, Ontario, Canada; University of the Pacific, UNITED STATES

## Abstract

Ions released from metal implants have been associated with adverse tissue reactions and are therefore a major concern. Studies with macrophages have shown that cobalt, chromium, and nickel ions can activate the NLRP3 inflammasome, a multiprotein complex responsible for the activation of caspase-1 (a proteolytic enzyme converting pro-interleukin [IL]-1β to mature IL-1β). However, the mechanism(s) of inflammasome activation by metal ions remain largely unknown. The objectives of the present study were to determine if, in macrophages: 1. caspase-1 activation and IL-1β release induced by metal ions are oxidative stress-dependent; and 2. IL-1β release induced by metal ions is NF-κB signaling pathway-dependent. Lipopolysaccharide (LPS)-primed murine bone marrow-derived macrophages (BMDM) were exposed to Co^2+^ (6–48 ppm), Cr^3+^ (100–500 ppm), or Ni^2+^ (12–96 ppm), in the presence or absence of a caspase-1 inhibitor (Z-WEHD-FMK), an antioxidant (L-ascorbic acid [L-AA]), or an NF-κB inhibitor (JSH-23). Culture supernatants were analyzed for caspase-1 by western blotting and/or IL-1β release by ELISA. Immunoblotting revealed the presence of caspase-1 (p20 subunit) in supernatants of BMDM incubated with Cr^3+^, but not with Ni^2+^ or Co^2+^. When L-AA (2 mM) was present with Cr^3+^, the caspase-1 p20 subunit was undetectable and IL-1β release decreased down to the level of the negative control, thereby demonstrating that caspase-1 activation and IL-1β release induced by Cr^3+^ was oxidative stress-dependent. ELISA demonstrated that Cr^3+^ induced the highest release of IL-1β, while Co^2+^ had no or limited effects. In the presence of Ni^2+^, the addition of L-AA (2 mM) also decreased IL-1β release, below the level of the negative control, suggesting that IL-1β release induced by Ni^2+^ was also oxidative stress-dependent. Finally, when present during both priming with LPS and activation with Cr^3+^, JSH-23 blocked IL-1β release, demonstrating NF-κB involvement. Overall, this study showed that while both Cr^3+^ and Ni^2+^ may be inducing inflammasome activation, Cr^3+^ is likely a more potent activator, acting through oxidative stress and the NF-κB signaling pathway.

## Introduction

Implantable metal alloys such as cobalt-chromium-molybdenum (CoCrMo) and stainless steel are widely used in medical devices, especially in hip and knee replacements [1]. However, material degradation through corrosion and wear mechanisms may compromise the structural integrity of the implants, and the biological effects of the wear and corrosion products are of great clinical concern [2,3]. Ion release from the alloy components is particularly concerning as elevated levels of metal ions have been reported in the serum, synovial fluid, and blood of patients with joint implants [4–6]. In addition, corrosion at the modular interfaces of joint implants have been associated with early adverse tissue reactions [7,8], including hypersensitivity reactions [9] and pseudotumors [10–12], and the long-term release of wear products can lead to chronic inflammation, and thereby periprosthetic osteolysis [13–15]. Overall, a better understanding of the molecular mechanisms leading to the inflammatory response to wear and corrosion products is necessary to develop potential therapeutic treatments as well as to facilitate the development of more compatible and durable biomaterial alloys.

Previous *in vitro* studies have shown that metal ions including Co^2+^, Cr^3+^, and Ni^2+^ can trigger the assembly of the nucleotide-binding oligomerization domain (NOD)-like receptor (NLR) family pyrin domain-containing protein 3 (NLRP3) inflammasome [16,17] in macrophages, the predominant cell type in periprosthetic tissues [14,18,19]. The NLRP3 inflammasome has become the most widely studied member of the inflammasome family since its discovery in 2002 [20] due to its capacity to be activated by a wide array of structurally dissimilar danger-associated molecular patterns (DAMP) and pathogen-associated molecular patterns (PAMP) [21–23]. It is a large multiprotein complex responsible for the release of mature interleukin (IL)-1β, a cytokine that plays a key role in inflammation, and it is tightly regulated through a two-step process referred to as priming and activation [24,25]. During the priming step, a PAMP binds to a toll-like receptor (TLR) type of pathogen recognition receptor (PRR) on the cell membrane surface [26], leading to the upregulation of pro-IL-1β and NLRP3 through the nuclear factor kappa-light-chain-enhancer of activated B cells (NF-κB) pathway [26]. During the activation step, a PAMP or DAMP causes NLRP3 to oligomerize and recruit the apoptosis-associated speck-like protein containing a C-terminal caspase-recruitment domain (ASC), which itself recruits pro-caspase-1 [25,27]. The recruitment of this pro-enzyme leads to autoproteolysis and assembly of the resulting subunits (p20 and p10) into enzymatically active caspase-1 that cleaves pro-IL-1β into mature IL-1β.

While previous studies have shown that Co^2+^, Cr^3+^, and Ni^2+^ can trigger the assembly of the NLRP3 inflammasome [16,17], the underlying mechanisms of activation remain largely unknown and constitute an active area of research in various fields including orthopaedics, periodontology [28], and allergology [17,29]. Basic immunological research has revealed that NLRP3 induction relies on NF-κB signaling [26], and that several DAMP can activate the NLRP3 inflammasome through reactive oxygen species (ROS) production [30], K^+^ efflux, lysosomal rupture, and/or spatial rearrangement of organelles [25,27]. Metal ions are known to adversely affect cellular function, as evidenced by their cytotoxicity [31–33]. More specifically, Co^2+^, Cr^3+^, and Ni^2+^ have been shown to induce an increase in ROS production in immune cells [34,35], as well as IL-1β release in macrophages [16,17] *in vitro*. Interestingly, IL-1β release induced by metal ions has been shown to decrease in the presence of an inhibitor of nicotinamide adenine dinucleotide phosphate (NADPH) oxidase [16], whose primary catalytic function is ROS production [36]. It is therefore possible that these metal ions activate the NLRP3 inflammasome through ROS production, following NF- κB activation.

The objectives of the present study were to determine if, in macrophages: 1. caspase-1 activation and IL-1β release induced by metal ions are oxidative stress-dependent; and 2. IL-1β release induced by metal ions is NF-κB signaling pathway-dependent. Overall, this study provides insights into the mechanisms of metal ion-induced NLRP3 inflammasome activation in macrophages.

## Materials and methods

### Metal ions

Stock solutions of Co^2+^, Cr^3+^, and Ni^2+^ were prepared fresh, as previously described [37]. Briefly, CoCl_2_•6H_2_O (≥99.5% purity; Fisher Scientific, Waltham, MA), CrCl_3_•6H_2_O (≥99.2% purity; Sigma-Aldrich, St Louis, MO) and NiCl_2_•6H_2_O (≥99.999% purity; Sigma-Aldrich) were dissolved in cell culture-grade water (Lonza, Walkersville, MD), and the solutions were sterilized by filtration through 0.2-μm pore size cellulose acetate syringe filters (VWR, Mississauga, ON).

### Cells

Bone marrow-derived macrophages (BMDM) were differentiated from bone marrow cells isolated from the femora, tibiae, and pelvic bones of 4- to 16-week-old female wild type C57BL/6J mice (The Jackson Laboratory, Bar Harbor, ME). Procedures were approved by the University of Ottawa Animal Care Committee (Protocol ME-2350). The University of Ottawa animal care and use program meets the Canadian Council on Animal Care (CCAC) guidelines and is licensed under the Province of Ontario Animals for Research Act. The mice were cared for and housed at the Animal Care Facility of the University of Ottawa, a specific-pathogen-free (SPF) facility. More specifically, mice (up to five per cage) were housed in individually ventilated cages (Sealsafe^®^; Techniplast, West Chester, PA) with 6-mm size corncob bedding (Envigo RMS, Indianapolis, IN), cotton fiber-based nesting material (Ancare, Bellmore, NY), and a shreddable refuge hut (Ketchum, Brockville, ON). The animals were maintained at 22°C and a relative humidity of 40% under a 12h-light:12h-dark photoperiod with *ad libitum* access to food (Teklad Global 18% Protein Rodent Diet; Envigo RMS) and water (purified by reverse osmosis and acidified to pH 2.5–3.0 with hydrochloric acid). Euthanasia was performed by CO_2_ gas asphyxiation followed by cervical dislocation. Euthanized mice were soaked with 70% (v/v) ethanol immediately prior to dissection.

After careful dissection and isolation of bones, bone marrow was flushed into a 100-μm nylon mesh cell strainer (Fisher Scientific) using a 27G x ½ inch needle-syringe (BD Biosciences, Durham, NC) filled with bone marrow cell differentiation medium consisting of Roswell Park Memorial Institute (RPMI) 1640 medium (Wisent, St-Bruno, QC) supplemented with 8% heat-inactivated ultra-low endotoxin fetal bovine serum (FBS, catalog no. NBSF-701; North Bio, Toronto, ON), 100 U/mL penicillin-streptomycin (Thermo Fisher Scientific, Waltham, MA), and 0.1% 2-mercaptoethanol (2-ME; Thermo Fisher Scientific). Strained bone marrow cells were centrifuged at 150 *g* for 10 min at room temperature (RT), and resuspended in the differentiation medium at 1.5 x 10^6^ cells/mL. Suspension dishes (100-mm diameter; Greiner Bio-One, Monroe, NC) were thinly coated with 50 μL of the differentiation medium containing 50 ng recombinant macrophage-colony stimulating factor (M-CSF; R&D Systems, Minneapolis, MN) using a disposable bacterial cell spreader (Excel Scientific, Victoriaville, CA), immediately prior to being seeded with 10 mL of cell suspension per dish. The dishes were then incubated for 6 days at 37°C in a humidified atmosphere of 95% air and 5% CO_2_.

At the end of the incubation, non-adherent cells were removed by rinsing the dishes with 5 mL of the differentiation medium, and mature adherent BMDM were harvested by pipetting with a 10-mL Class A volumetric pipette (SIBATA Scientific Technology, Saitama, Japan). The collected BMDM were centrifuged at 300 *g* for 10 min at RT and resuspended at 1 x 10^6^ cells/mL in growth medium (differentiation medium without 2-ME). Twenty-four well tissue culture-treated plates (Greiner Bio-One) were then seeded with 0.3 mL of cell suspension per well and incubated 4h under cell culture conditions to allow cell attachment. Unless otherwise stated, at the end of the incubation, the culture supernatants were discarded and the adherent BMDM in each well were primed by exposure to 500 ng/mL lipopolysaccharide (LPS; Sigma-Aldrich) for 6h under cell culture conditions.

### Caspase-1 inhibition

At the end of the priming incubation, the medium containing LPS was replaced with growth medium containing 0 or 20 μM Z-WEHD-FMK (catalog no. FMK002; R&D Systems), an irreversible caspase-1 inhibitor, and the cells were incubated 1h under cell culture conditions. Co^2+^ (6 to 48 ppm final concentrations), Cr^3+^ (100 to 500 ppm), Ni^2+^ (12 to 96 ppm), nigericin (5 μM; positive control), or cell-culture grade water (ion solvent used as the negative control) was then added to the culture supernatants and the cells were incubated an additional 18 to 24h. At the end of the incubation, supernatants were collected, centrifuged at 300 *g* for 10 min at 4°C, snap-frozen in liquid nitrogen, and stored at -80°C for later analysis of IL-1β release by enzyme-linked immunosorbent assay (ELISA).

Freshly thawed culture supernatants were gently mixed and concentrations of IL-1β were measured by ELISA using a commercial kit (IL-1 beta Mouse Uncoated ELISA Kit; Thermo Fisher Scientific), as per the manufacturer’s instructions. Absorbance measurements were performed at 450 nm using a hybrid microplate reader (Synergy™ 4; BioTek, Winooski, VT). Absorbance at the reference wavelength of 570 nm was subtracted from the measurements. The nominal minimum concentration of IL-1β detectable by ELISA was 8 pg/mL, as per the manufacturer’s specifications.

### Oxidative stress inhibition

At the end of the priming incubation, the medium containing LPS was replaced with growth medium containing 0 or 2 mM L-ascorbic acid (L-AA, catalog no. A4544-100G; Sigma-Aldrich), an antioxidant, and the cells were incubated 1h under cell culture conditions. Co^2+^ (18 ppm final concentration), Cr^3+^ (300 ppm), Ni^2+^ (48 ppm), nigericin (5 μM; positive control), or cell-culture grade water (negative control) was then added to the culture supernatants and the cells were incubated an additional 18 to 24h. These ion concentrations were selected because they induced the highest IL-1β release when analyzing caspase-1 inhibition. At the end of the incubation, supernatants were collected, centrifuged at 300 *g* for 10 min at 4°C, snap-frozen in liquid nitrogen, and stored at -80°C for later analysis of caspase-1 by western blotting (as detailed below) and IL-1β release by ELISA (as described above).

Freshly thawed culture supernatants were vortex-mixed, and protein determination was performed using the bicinchoninic acid colorimetric assay with bovine serum albumin (BSA) as the protein standard (Thermo Fisher Scientific). Absorbance (562 nm) was measured using a hybrid microplate reader (Synergy™ 4; BioTek). Aliquots (30 μg protein) of the culture supernatants were analyzed by sodium dodecyl sulfate (SDS)-polyacrylamide gel electrophoresis (PAGE) using precast mini-format tris-glycine gradient (8–16%) gels (Bio-Rad, Hercules, CA). Pre-stained protein molecular weight standards (Bio-Rad) were used. For western blotting, proteins were electrotransferred onto a 0.45-μm pore size polyvinylidene fluoride (PVDF) membrane (Millipore, Billerica, MA). The blotted membrane was reversibly stained for total protein with 0.1% (w/v) Ponceau S (catalog no. BP103-10; Fisher Scientific) in 5% (v/v) acetic acid (Fisher Scientific). Tris-buffered saline (TBS; 20 mM Tris base [Fisher Scientific], 130 mM NaCl [Fisher Scientific], pH 7.6) containing 5% immunoanalytical-grade non-fat dry milk (Blotto^®^; Rockland Inc., Limerick, PA) and TBS were used as the blocking and antibody dilution buffer, respectively. The primary antibody, a mouse anti-caspase-1 p20 subunit monoclonal antibody (catalog no. AG-20B-0042-C100; Adipogen, San Diego, CA), and the secondary antibody, a polyclonal anti-mouse IgG horseradish peroxidase (HRP) conjugate (catalog no. W4021; Promega, Madison, WI), were used at a dilution of 1:1000 and 1:5000, respectively. Chemiluminescence detection was performed using enhanced chemiluminescent (ECL) HRP substrate (Millipore), as per the manufacturer’s instructions, and the blots were imaged using a near-infrared fluorescence/chemiluminescence imaging system (Odyssey Fc; LI-COR Biosciences, Lincoln, NE).

### NF-κB (p65) transcription factor blocking

At the end of the incubation for cell attachment, the culture supernatants were replaced with growth medium containing 0 or 60 μM JSH-23 (i.e., 4-methyl-N1-(3-phenylpropyl)-1,2-benzenediamine, catalog no. 15036; Cayman Chemical, Ann Arbor, MI), an NF-κB transcription factor inhibitor, and the cells were incubated 1h under cell culture conditions. LPS (500 ng/mL final concentration) was then added to each well, and the cells were incubated 6h under cell culture conditions. At the end of this incubation, the culture supernatants were replaced with growth medium containing 0 or 60 μM JSH-23, and the cells were incubated an additional 1h. Cr^3+^ (300 ppm final concentration), nigericin (5 μM; positive control), or cell culture-grade water (negative control) was then added to the culture supernatants and the cells were incubated an additional 18 to 24h. In total, four JSH-23 conditions were tested: no JSH-23, JSH-23 present exclusively during the priming incubation with LPS, JSH-23 present exclusively during the activation incubation with Cr^3+^ or nigericin, and JSH-23 present during both incubations. At the end of the 18 to 24h incubation, supernatants were collected, centrifuged at 300 *g* for 10 min at 4°C, snap-frozen in liquid nitrogen, and stored at -80°C for IL-1β measurements by ELISA, as described above.

### Cell mortality assessment

At the end of the experiments, adherent cells were washed with 0.5 mL/well of ice-cold Dulbecco’s phosphate buffered saline (DPBS; Sigma-Aldrich), incubated 15 min at RT in 300 μL of an enzymatic cell detachment solution (Accutase^®^; Thermo Fisher Scientific), and detached by gentle pipetting using a 1-mL manual single-channel pipette (Mettler Toledo, Columbus, OH). The cell suspensions were transferred into 2-mL untreated polystyrene culture tubes (Axygen Scientific, Union City, CA), and cell mortality was analyzed by dye-exclusion hemocytometry under phase contrast microscopy using trypan blue (0.04% [w/v] final concentration; Sigma-Aldrich) and an improved Neubauer hemocytometer (Hausser Scientific, Horsham, PA).

### Statistical analysis

Statistical analysis was performed in SPSS v24.0 (IBM, Armonk, NY) using additive two-way analysis of variance (ANOVA) with ion concentration as a fixed effect and experiment as a random effect, and Tukey-Kramer post-hoc pairwise tests. *p*<0.05 was considered significant.

## Results

### Effects of caspase-1 inhibitor (Z-WEHD-FMK) on IL-1β release

**Fig 1:**
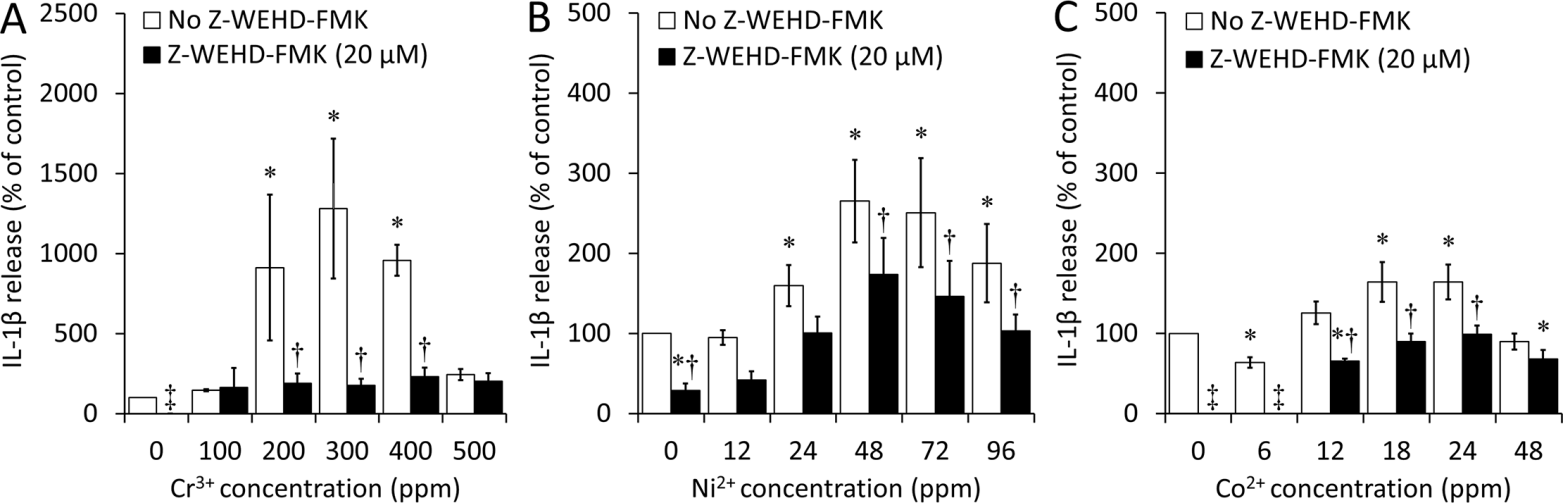
**Interleukin-1β (IL-1β) release by bone marrow-derived macrophages (BMDM) after exposure to (A) Cr**^**3+**^**, (B) Ni**^**2+**^**, and (C) Co**^**2+**^**, with or without Z-WEHD-FMK.** Cells were incubated under cell culture conditions with the indicated concentrations of ions in the presence or absence of Z-WEHD-FMK (20 μM), a caspase-1 inhibitor, for 18 to 24h following a 6h priming incubation with lipopolysaccharide (LPS; 500 ng/mL). IL-1β release was analyzed by ELISA and is expressed as a percentage of the release in the negative control (cells with no ions and no Z-WEHD-FMK). An asterisk (*) indicates a significant difference (*p*<0.05) between a given ion concentration with or without Z-WEHD-FMK and the negative control. A dagger (†) indicates a significant difference (*p*<0.05) between the results obtained with and without Z-WEHD-FMK at a given ion concentration. A double dagger (‡) indicates that the measurement was below the detection threshold. Data are presented as means ± SEM of 3–5 independent experiments performed in triplicate.

ELISA results revealed the highest increase in IL-1β release with Cr^3+^, up to 1280% with 300 ppm (*p*<0.001), relative to the negative control (cells with no ions and no Z-WEHD-FMK) (Fig 1A). The increase was not significant with 500 ppm Cr^3+^, likely due to the higher toxicity of Cr^3+^ at this elevated concentration. IL-1β release also increased with Ni^2+^, up to 265% with 48 ppm (*p*<0.001), relative to the negative control (Fig 1B). As with Cr^3+^, the release decreased with higher Ni^2+^ concentrations, likely reflecting a higher toxicity of Ni^2+^ as concentration increases. Finally, incubation with Co^2+^ revealed a small but statistically significant increase in IL-1β release, up to 164% with 18 ppm and 24 ppm (*p*<0.001), relative to the negative control (Fig 1C). The increase was not significant with 48 ppm, once again likely reflecting the higher toxicity of Co^2+^ at this elevated concentration. Interestingly, a decrease of 36% was observed with 6 ppm Co^2+^ (*p*<0.001).

The presence of Z-WEHD-FMK (20 μM) induced a decrease of 76% to 86% in IL-1β release with 200 to 400 ppm Cr^3+^ (*p*<0.001 in all cases) (Fig 1A), down to levels similar to that of the negative control. It also induced a decrease of 35% to 45% with 48 ppm Ni^2+^ or higher (*p*≤0.002 in all cases) (Fig 1B). Finally, this caspase-1 inhibitor induced a decrease with 6 ppm Co^2+^, down to a level below the detection threshold, and a decrease of 40% to 48% with 12 to 24 ppm Co^2+^ (*p*<0.001 in all cases), down to levels similar to that of the negative control (Fig 1C).

Trypan blue dye-exclusion analysis revealed an ion concentration-dependent increase in the percentages of dead cells, up to 52% with 500 ppm Cr^3+^ (*p*<0.001) (Panel A in S1 Fig), 70% with 96 ppm Ni^2+^ (*p*<0.001) (Panel C in S1 Fig), and 71% with 48 ppm Co^2+^ (*p*<0.001) (Panel E in S1 Fig), relative to the negative control (cells with no ions and no Z-WEHD-FMK). The total number of cells (live and dead) decreased by up to 30% with 400 ppm Cr^3+^ (Panel B in S1 Fig), 50% with 96 ppm Ni^2+^ (Panel D in S1 Fig), and 28% with 48 ppm Co^2+^ (Panel F in S1 Fig) (*p*<0.001 in all cases). Notably, the presence of 20 μM Z-WEHD-FMK did not have a significant effect on the percentage of dead cells with Cr^3+^ and Ni^2+^ (Panels A and C in S1 Fig), and decreased it with 24 ppm Co^2+^ (from 56% to 51%; *p* = 0.025) and 48 ppm Co^2+^ (from 71% to 65%; *p* = 0.006) (Panel E in S1 Fig). Finally, the presence of 20 μM Z-WEHD-FMK also did not induce any significant differences in the total number of cells, except with 200 ppm Cr^3+^ (decreased from 89% to 63%, relative to the negative control; *p*<0.001) (Panel B in S1 Fig). Overall, Z-WEHD-FMK was considered to have minimal toxic effects in the conditions analyzed.

### Effects of antioxidant (L-ascorbic acid) on caspase-1 activation and IL-1β release

**Fig 2:**
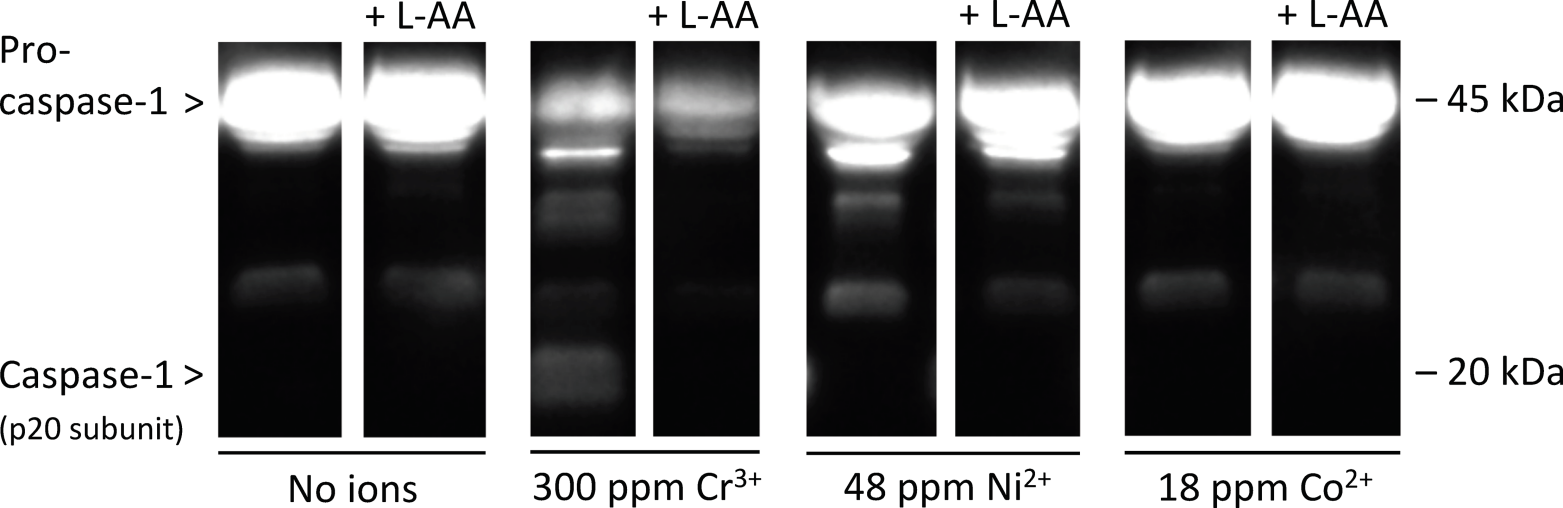
Caspase-1 activation in bone marrow-derived macrophages (BMDM) after exposure to Cr^3+^, Ni^2+^, and Co^2+^, with or without L-ascorbic acid (L-AA). Cells were incubated under cell culture conditions with the indicated concentrations of ions in the presence or absence of L-AA (2 mM), an antioxidant, for 18 to 24h following a 6h priming incubation with lipopolysaccharide (LPS; 500 ng/mL). Culture supernatants were analyzed for the presence of caspase-1 (as indicated by the detection of its p20 subunit) by western blotting. The immunoblot presented is representative of three independent experiments.

Immunoblotting results revealed the presence of the caspase-1 (as indicated by the detection of its p20 subunit) in the supernatants of cells incubated with 300 ppm Cr^3+^, but not with 48 ppm Ni^2+^ or 18 ppm Co^2+^ (Fig 2). Additionally, pro-caspase-1 was less abundant in BMDM exposed to Cr^3+^ compared to BMDM exposed to Ni^2+^ or Co^2+^. Importantly, the p20 subunit, observed with 300 ppm Cr^3+^, was undetectable when L-AA (2 mM) was also present.

**Fig 3:**
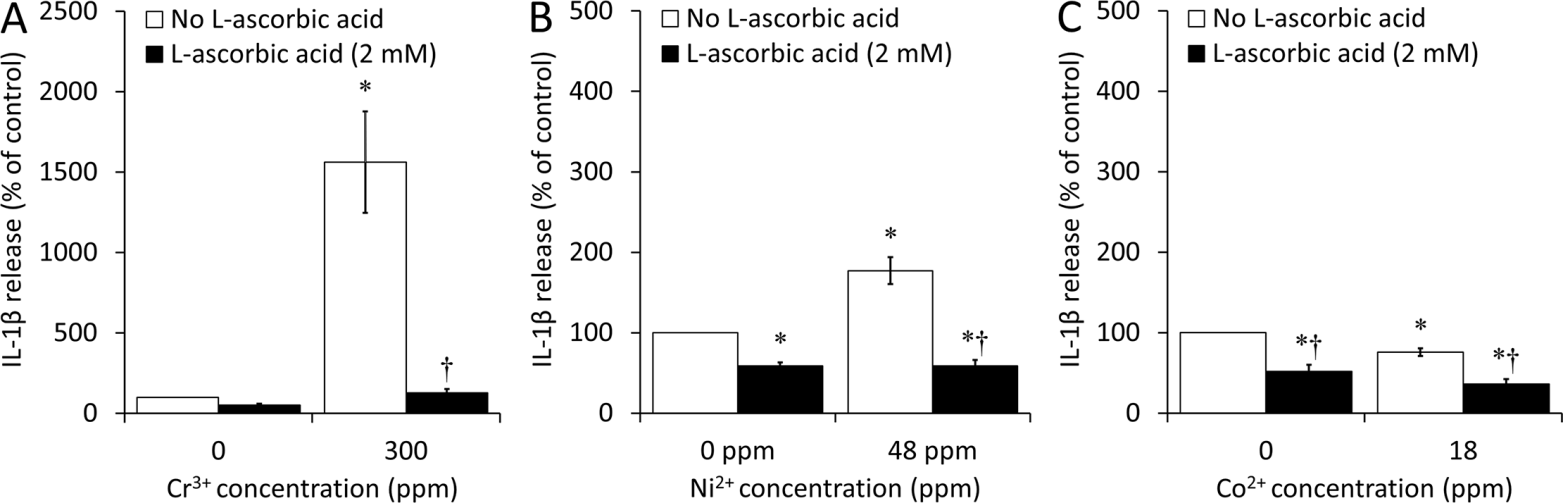
**Interleukin-1β (IL-1β) release by bone marrow-derived macrophages (BMDM) after exposure to (A) Cr**^**3+**^**, (B) Ni**^**2+**^**, and (C) Co**^**2+**^**, with or without L-ascorbic acid (L-AA).** Cells were incubated under cell culture conditions with the indicated concentrations of ions in the presence or absence of L-AA (2 mM), an antioxidant, for 18 to 24h following a 6h priming incubation with lipopolysaccharide (LPS; 500 ng/mL). IL-1β release was analyzed by ELISA and is expressed as a percentage of the release in the negative control (cells with no ions and no L-AA). An asterisk (*) indicates a significant difference (*p*<0.05) between a given ion concentration with or without L-AA and the negative control. A dagger (†) indicates a significant difference (*p*<0.05) between the results obtained with and without L-AA at a given ion concentration. Data are presented as means ± SEM of 3 independent experiments performed in triplicate.

ELISA results showed that the presence of L-AA (2 mM) induced a decrease of 92% in IL-1β release with 300 ppm Cr^3+^ (*p*<0.001; Fig 3A), as well as a decrease of 67% with 48 ppm Ni^2+^ (*p*<0.001; Fig 3B), down to or below the level of the negative control (cells with no ions and no L-AA), respectively. Co^2+^ (18 ppm) did not induce significant IL-1β increase (Fig 3C).

Notably, while the presence of 2 mM L-AA did not induce any significant differences in the percentage of dead cells with 300 ppm Cr^3+^ and 48 ppm Ni^2+^ (Panels A and C in S2 Fig, respectively), it induced a small but significant increase in the percentage of dead cells with 18 ppm Co^2+^ (from 39% to 45%; *p* = 0.012) (Panel E in S2 Fig). Finally, while the presence of 2 mM L-AA induced a small but significant decrease in the total number of cells with 300 ppm Cr^3+^ (from 65% to 52%, relative to the negative control; *p* = 0.026) (Panel B in S2 Fig), the differences were not significant with 48 ppm Ni^2+^ and 18 ppm Co^2+^ (Panels D and F in S2 Fig, respectively). Overall, L-AA was considered to have minimal toxic effects in the conditions analyzed.

### Effects of NF-κB inhibitor (JSH-23) on IL-1β release

**Fig 4:**
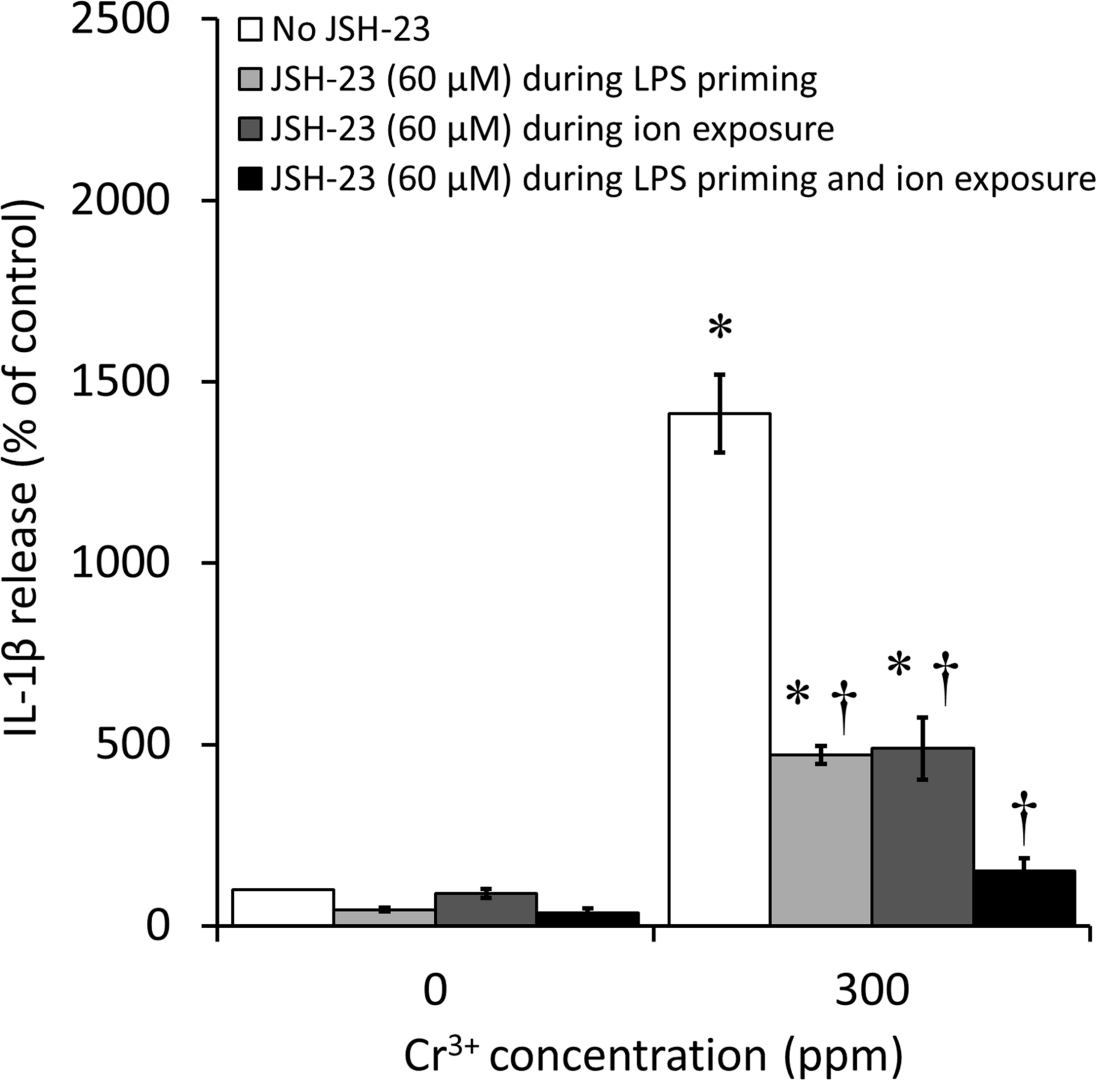
Interleukin-1β (IL-1β) release by bone marrow-derived macrophages (BMDM) after exposure to Cr^3+^, with or without JSH-23. Cells were incubated under cell culture conditions with or without Cr^3+^ (300 ppm) in the presence or absence of JSH-23 (60 μM), an NF-κB inhibitor, for 18 to 24h following a 6h priming incubation with lipopolysaccharide (LPS; 500 ng/mL). In total, four JSH-23 conditions were tested: no JSH-23 (white columns), JSH-23 present exclusively during the priming incubation with LPS (light gray columns), JSH-23 present exclusively during the activation incubation with Cr^3+^ (dark gray columns), and JSH-23 present during both incubations (black columns). IL-1β release was analyzed by ELISA and is expressed as a percentage of the release in the negative control (cells with no ions and no JSH-23). An asterisk (*) indicates a significant difference (*p*<0.05) between a given ion concentration with or without JSH-23 and the negative control. A dagger (†) indicates a significant difference (*p*<0.05) between the results obtained with and without JSH 23 at a given ion concentration. Data are presented as means ± SEM of 3 independent experiments performed in triplicate.

ELISA results revealed that when present during both the 6h LPS-priming incubation and the 18 to 24h incubation with Cr^3+^, JSH-23 (60 μM) induced a decrease of 89% in IL-1β release (*p*<0.001), down to the level of the negative control (cells with no ions and no JSH-23) (Fig 4). When present during either the 6h LPS-priming incubation or the 18 to 24h incubation with Cr^3+^, JSH-23 (60 μM) induced only a partial decrease of 67% and 65% in IL-1β release (*p*<0.001 in both cases), respectively, and the levels remained higher than that of the negative control. The presence of JSH-23 did not have a significant effect in the negative control.

Notably, when present during both the LPS priming incubation and the ion incubation, 60 μM JSH-23 induced a small but significant increase in the percentage of dead cells in the negative control (from 6% to 12%; *p*<0.001) (Panel A in S3 Fig), along with a significant decrease in the total number of cells (from 100% to 84%; *p*<0.001) (Panel B in S3 Fig). However, it did not induce any significant differences in the percentage of dead cells nor in the total number of cells with 300 ppm Cr^3+^ (Panels A and B in S3 Fig, respectively). Overall, JSH-23 was considered to have minimal toxic effects in the conditions analyzed.

## Discussion

The present study analyzed the effects of Co^2+^, Cr^3+^ and Ni^2+^ on caspase-1 activation and IL-1β release (a probable indicator of inflammasome assembly) in BMDM. This study focused on the effects of Co^2+^, Cr^3+^ and Ni^2+^ because these ions are released from wear and corrosion of CoCrMo and stainless steel alloys (widely used in medical devices) and are a major cause for concern [7–12]. The ranges of ion concentrations were based on: 1. past *in vitro* studies analyzing metal ion-induced macrophage cytotoxicity and cytokine release [31–33,38]; 2. the assumption that macrophage stimulation is likely to require higher concentrations of stimulating agent *in vitro* than it does *in vivo* (where cells are exposed to multiple stimulating factors simultaneously); and 3. the assumption that the concentration of these ions is higher in periprosthetic tissues than in body fluids where their concentrations are in the ppb range [32].

Macrophages have been shown to be the predominant cell type in periprosthetic tissues [14,18,19]. As part of the innate immune system front line of defense, they interact with wear particles and metal ions and secrete pro-inflammatory cytokines [39]. While the subsequent chain of events leading to periprosthetic osteolysis is well described in the literature [13,40,41], the molecular mechanisms of macrophage activation by wear particles and metal ions still remain largely unknown. Murine BMDM were used in this study because of their greater physiological relevance to *in vivo* systems compared to cell lines [42]. In addition, murine bone marrow provides a high yield of BMDM and these macrophages are easier to generate than primary murine macrophages from other sources such as the peritoneal cavity or pulmonary alveoli [43–45]. Finally, in addition to being the macrophage model of choice in most immunological studies [45], BMDM have been previously used to investigate Ni^2+^-induced inflammasome activation [17].

Results revealed a concentration-dependent increase in BMDM mortality with the three metal ions analyzed. Overall, Co^2+^ and Ni^2+^ were more toxic than Cr^3+^, in agreement with previous studies with J774 macrophages [31,38]. The presence of the different inhibitors (Z-WEHD-FMK, L-ascorbic acid, JSH-23) had minimal toxic effect at the concentrations analyzed (see Supporting Information).

Results also showed a metal ion concentration-dependent increase in IL-1β release with Cr^3+^ and with Ni^2+^ (albeit to a lower extent), while Co^2+^ had no or limited effects. Indeed, while the results of the experiments analyzing the effects of caspase-1 inhibitor showed a small but statistically significant IL-1β increase with Co^2+^ (up to 164%, relative to the negative control), the release was not significant when analyzing the effects of L-AA, revealing inter-animal variability (100% of the animals responded positively to Cr^3+^ and Ni^2+^ whereas less than 50% of the animals responded positively to Co^2+^), and suggesting that the small increase that was initially observed was not biologically significant. Interestingly, IL-1β release was highest with Cr^3+^ (almost 5 times higher than with Ni^2+^) and was inhibited by the presence of Z-WEHD-FMK, a cell-permeable fluoromethyl ketone (FMK)-derivatized peptide acting as a specific inhibitor of caspase-1. This strongly suggests that IL-1β release was dependent on pro-IL-1β cleavage by caspase-1. Because caspase-1 catalytically auto-activates when assembled by the NLRP3 inflammasome complex [24,25,46], results suggest that Cr^3+^ is an activator of the inflammasome. The much lower IL-1β release with Ni^2+^, together with the apparent absence of caspase-1 (p20 subunit) (possibly because the signal was below the detection threshold), suggests that Cr^3+^ is a more potent activator of the NLRP3 inflammasome than Ni^2+^. In addition, while the level of IL-1β with Ni^2+^ in the presence of caspase-1 inhibitor was not statistically different from that of the negative control (likely because of the relatively large SEM), it remained at 174% and 146% of the negative control with 48 ppm and 72 ppm Ni^2+^, respectively, suggesting that the inhibition of IL-1β by the caspase-1 inhibitor might not be complete and that Ni^2+^ may therefore be acting through additional molecular mechanisms. Finally, Co^2+^ appeared to have no or limited effects on the inflammasome pathway.

Interestingly, past studies have shown that the three metal ions analyzed in the present study induce IL-1β release and suggested NLRP3 inflammasome activation as the most probable cause [16,17]. For example, Caicedo et al. [16] showed that Co^2+^, Cr^3+^, and Ni^2+^ led to IL-1β release in isolated primary human macrophages, and that this release was caspase-1- and NLRP3 inflammasome-dependent. Notably, the authors reported levels of IL-1β that were significantly higher than the ones observed in the present study, which may be explained by differences in cell types (human vs. murine), and/or differences in experimental design. Additionally, Li et al. [17] reported that Ni^2+^ induce IL-1β release via the NLRP3 inflammasome pathway in different cell types, including BMDM. The levels of IL-1β release by BMDM were the lowest, but were overall comparable to those with Ni^2+^ in the present study. Interestingly, while Li et al. also demonstrated the presence of the caspase-1 p20 subunit in lysates of phorbol 12-myristate 13-acetate (PMA)-primed THP-1 cells exposed to an equivalent of 90 ppm Ni^2+^ (suggesting inflammasome activation), the p20 subunit was not detected in the supernatants of BMDM exposed to 48 ppm Ni^2+^ in the present study. The differences in the Ni^2+^ concentrations are unlikely to explain the differences in the p20 subunit detection since IL-1β release was significantly lower with 96 ppm Ni^2+^ than with 48 ppm in the present study. However, once again, differences in cell types (immortalized human cells in Li et al. study vs. primary BMDM in the present study), and/or differences in experimental design may explain the differences in the results. Notably, culture supernatants were analyzed in the present study since the bulk of stable caspase-1 has been reported to be secreted following inflammasome activation [47,48]. Assuming that the inflammasome is assembled in BMDM in response to Ni^2+^ (as suggested by the detection of IL-1β, albeit at lower levels than with Cr^3+^), it is it possible that the absence of the p20 subunit detection by western blotting may have been due to a signal below the detection threshold. Finally, some studies [28,29] reported no IL-1β release by THP-1 cells in response to Co^2+^ and Cr^3+^. While results with Co^2+^ are in agreement with those from the present study, differences with Cr^3+^ may be explained by differences in the ion concentration (higher in the present study). Overall, the reported levels of IL-1β release are highly variable across studies, likely due to differences in cell types, cell culture reagents, and even methodologies, including ion solution preparation.

The differences in the ion-induced IL-1β release observed in the present study (Fig 1) suggest that Cr^3+^ may be an important contributor to aseptic inflammation *in vivo*. However, Ni^2+^ and Co^2+^ may also contribute to aseptic inflammation through different mechanisms and/or may elicit different immunological responses. For example, Ni^2+^, thought to be the most potent immunological sensitizing metal ion [49], has been associated with hypersensitivity reactions [50,51]. The elevated mass percentage of nickel in stainless steel alloys such as 316L (10–15% compared to <2% in CoCrMo alloys) [52], coupled with the propensity of stainless steels for pitting, crevice, and stress corrosion [53], may therefore contribute to the hypersensitivity-type adverse local tissue reactions (ALTR) often reported surrounding these alloys. Nevertheless, the potential for an implantable alloy to generate an adverse response is complex and multifactorial, involving not only material and compositional concerns but also tribological factors. For example, larger and irregularly-shaped CoCrMo particles have been reported to increase inflammasome activation in macrophages [54].

The NLRP3 inflammasome has been shown to be activated by a wide array of danger patterns, including pathogen-derived proteins [23], inorganic silica crystals, and aluminum salts [22,55,56]. Indeed, the NLRP3 inflammasome has been identified as a potential sensor of metabolic danger or stress [57]. Because of the significant structural differences between these activators, the NLRP3 inflammasome is not thought to bind directly to its activators or be dependent on a single activation pathway [25]. Instead, NLRP3 inflammasome activation is thought to be the result of potentially concomitant signaling pathways, including ROS production [30], K^+^ efflux, lysosomal rupture, and/or spatial rearrangement of organelles [25,27]. Studies have shown that Cr^3+^ lead to the production of ROS such as superoxide ions, hydrogen peroxide, and hydroxyl radicals via redox cycling [58]. Additionally, metal ions may be causing a dysfunction of the mitochondria, which are responsible for most ROS production [25]. It has also been previously demonstrated that metal ion-induced inflammasome activation was dependent on NADPH-generated ROS in THP-1 cells [16]. It is therefore possible that metal ions induce inflammasome activation via ROS production. Remarkably, results of the present study showed that the presence of L-AA, an inhibitor of ROS production, inhibited caspase-1 activation (no detection of p20 subunit) as well as IL-1β release by BMDM exposed to Cr^3+^, suggesting the involvement of ROS in Cr^3+^-induced inflammasome activation and subsequent production of active caspase-1.

To determine the role of the NF-κB pathway in Cr^3+^-induced inflammasome activation, BMDM were cultured in the presence of JSH-23 during the priming incubation (LPS exposure), activation incubation (ion exposure), or both. JSH-23 is an inhibitor of the NF-κB transcription factor and, unlike most NF-κB inhibitors which act by preventing IκB kinase (IKK) from degrading IκBα (inhibitor of NF-κB alpha) and releasing active NF-κB, JSH-23 acts by preventing nuclear translocation of active NF-κB [59]. It is well established that inflammasome priming relies on the NF-κB pathway [25–27,46,60], and Bauernfeind et al. [26] reported that NLRP3 induction (priming) and caspase-1 activation were dose-dependently inhibited by an NF-κB inhibitor (Bay11-7082) in C57BL/6J immortalized wild-type splenic macrophages. Interestingly, the present study showed that the presence of 60 μM JSH-23 exclusively during the priming incubation only partially reduced Cr^3+^-induced IL-1β release, while 30 μM of JSH-23 completely blocked the expression of pro-IL-1β transcripts in RAW 264.7 exposed to LPS [59]. The effects of JSH-23 concentrations higher than 60 μM were not tested in the present study due to cytotoxicity concerns. It is possible that the partial, as opposed to complete, inhibition of IL-1β may have been due to residual LPS remaining during the activation incubation (when JSH-23 was not present), which may have induced some priming. Indeed, despite the removal of LPS-containing media and rinsing of the cells prior to ion exposure, residual LPS may have remained on cell membranes and/or culture substrates. Surprisingly, the presence of JSH-23 during exclusively the activation incubation (with the ions) partially decreased Cr^3+^-induced IL-1β release, suggesting a role for NF-κB during the activation of the inflammasome. While NLRP3 inflammasome activation has been reported to have regulatory effects on NF-κB activation [61], the regulation of NLRP3 activation by NF-κB remains poorly understood. In the present study, the role of NK-κB during the activation may have been related to residual LPS from the priming incubation, prolonging the production of NLRP3 and pro-IL-1β past the priming incubation. The presence of JSH-23 during the activation incubation would then suppress this prolonged production of NLPR3 and pro-IL-1β, thereby decreasing mature IL-1β release. Nevertheless, the potential effects of NF-κB on the inflammasome activation cannot be excluded. Interestingly, the presence of JSH-23 throughout both the inflammasome priming and activation incubations led to a significant decrease in Cr^3+^-induced IL-1β release, down to a level similar to that of the negative control (cells with no ions and no JSH-23). This suggests that metal ion-induced inflammasome assembly is dependent on the NF-κB pathway, though the relative involvement of this pathway during the priming and activation incubations remains to be further investigated.

Notably, priming of the BMDM with LPS prior to metal ion exposure was necessary to detect IL-1β release, which is in agreement with the *in vitro* model of macrophage priming with a TLR4 ligand [62,63]. Greenfield et al. [64,65] also demonstrated that endotoxins adherent to titanium particles greatly enhanced the particle biological activity via TLR2 and TLR4 binding in both RAW 264.7 macrophages and BMDM, and suggested that endogenous alarmins caused by particle-induced damage are not sufficient to activate TLR. Furthermore, Samelko et al. [66] recently reported that the IL-1β release by murine peritoneal macrophages exposed to CoCr particles more than doubled when LPS (a TLR-4 ligand) was present. However, in contrast with the present study, the addition of LPS was not necessary to observe IL-1β release. This difference may be due to differences in cell types (peritoneal macrophages in Samelko et al. vs. BMDM in the present study), or due to potential differences in the FBS used in the culture medium (unspecified grade of FBS in Samelko et al. vs. ultra-low endotoxin FBS in the present study). In any case, it should be noted that *in vivo*, priming may also be achieved by endogenous factors or alarmins [67–69], by ions directly binding to a TLR or through PAMP (including LPS) originating from a subclinical bacterial biofilm on the implant material surface [70,71].

In conclusion, this study demonstrated that Cr^3+^ and Ni^2+^ induced IL-1β release (probable indicator of inflammasome assembly) by BMDM. Nevertheless, the much higher release of IL-1β with Cr^3+^ and the absence of detectable caspase-1 (p20 subunit) with Ni^2+^ suggest that Cr^3+^ is a more potent activator of this pathway. Co^2+^, on the other hand, appeared to have either no or limited effects on the activation of this pathway. Finally, results showed that caspase-1 activation by Cr^3+^ was oxidative stress-dependent, and that the NF-κB signaling pathway was involved in IL-1β release. Overall, this study provides insights into the mechanisms of metal ion-induced NLRP3 inflammasome activation in macrophages, which may eventually help the development of pharmaceutical approaches to modulate the inflammatory response to metal ions, and thereby increase implant longevity.

## Supporting information

S1 Fig **Mortality of bone marrow-derived macrophages (BMDM) after exposure to Cr**^**3+**^**, Ni**^**2+**^**, or Co**^**2+**^**, with or without Z-WEHD-FMK: (A, C and E) Percentages of dead cells; (B, D and F) Total numbers of cells (viable and dead).** Cells were incubated under cell culture conditions with the indicated concentrations of ions in the presence or absence of Z-WEHD-FMK (20 μM), a caspase-1 inhibitor, for 18 to 24h following a 6h priming incubation with lipopolysaccharide (LPS; 500 ng/mL). Cells were counted by hemocytometry and dead cells were identified using the trypan blue dye-exclusion method. The total numbers of cells (viable and dead) were expressed as a ratio of the total number of cells in the negative control (cells with no ions and no Z-WEHD-FMK). An asterisk (*) indicates a significant difference (*p*<0.05) between a given ion concentration with or without Z-WEHD-FMK and the negative control. A dagger (†) indicates a significant difference (*p*<0.05) between the results obtained with and without Z-WEHD-FMK at a given ion concentration. Data are presented as means ± SEM of 3–4 independent experiments performed in triplicate.
(TIF)

S2 Fig **Mortality of bone marrow-derived macrophages (BMDM) after exposure to Cr**^**3+**^**, Ni**^**2+**^**, or Co**^**2+**^**, with or without L-AA: (A, C and E) Percentages of dead cells; (B, D and F) Total numbers of cells (viable and dead).** Cells were incubated under cell culture conditions with the indicated concentrations of ions in the presence or absence of L-AA (2 mM), an antioxidant, for 18 to 24h following a 6h priming incubation with lipopolysaccharide (LPS; 500 ng/mL). Cells were counted by hemocytometry and dead cells were identified using the trypan blue dye-exclusion method. The total numbers of cells (viable and dead) were expressed as a ratio of the total number of cells in the negative control (cells with no ions and no L-AA). An asterisk (*) indicates a significant difference (*p*<0.05) between a given ion concentration with or without L-AA and the negative control. A dagger (†) indicates a significant difference (*p*<0.05) between the results obtained with and without L-AA at a given ion concentration. Data are presented as means ± SEM of 3 independent experiments performed in triplicate.
(TIF)

S3 Fig **Mortality of bone marrow-derived macrophages (BMDM) after exposure to Cr**^**3+**^ **with or without JSH-23: (A) Percentages of dead cells; (B) Total numbers of cells (viable and dead).** Cells were incubated under cell culture conditions with or without Cr^3+^ (300 ppm) in the presence or absence of JSH-23 (60 μM), an NF-κB inhibitor, for 18 to 24h following a 6h priming incubation with 500 ng/mL of lipopolysaccharide (LPS). In total, four JSH-23 conditions were tested: no JSH-23 (white columns), JSH-23 present exclusively during the priming incubation with LPS (light gray columns), JSH-23 present exclusively during the activation incubation with Cr^3+^ (dark gray columns), and JSH-23 present during both incubations (black columns). Cells were counted by hemocytometry and dead cells were identified using the trypan blue dye-exclusion method. The total numbers of cells (viable and dead) were expressed as a ratio of the total number of cells in the negative control (cells with no ions and no JSH-23). An asterisk (*) indicates a significant difference (*p*<0.05) between a given ion concentration with or without JSH-23 and the negative control (cells with no ions and no JSH-23). A dagger (†) indicates a significant difference (*p*<0.05) between the results obtained with and without JSH 23 at a given ion concentration. Data are presented as means ± SEM of 3 independent experiments performed in triplicate.
(TIF)
