# Supplementary figures and images for: Effects of metal ions on caspase-1 activation and interleukin-1β release in murine bone marrow-derived macrophages
Source: PLoS One. 2018 Aug 23;13(8):e0199936. doi: 10.1371/journal.pone.0199936 (PMC6107125; doi:10.1371/journal.pone.0199936)

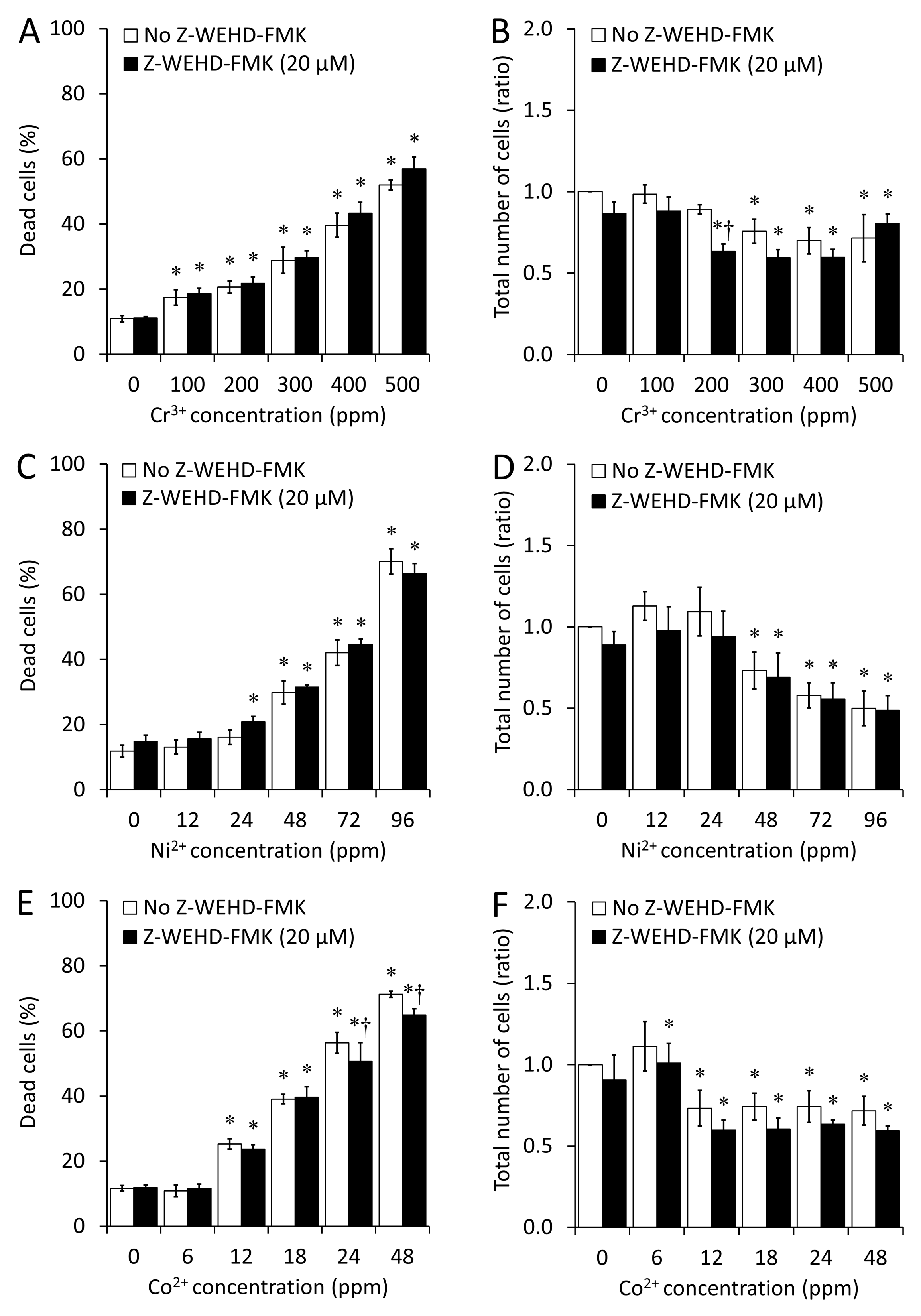

Supplement: S1 Fig — Mortality of bone marrow-derived macrophages (BMDM) after exposure to Cr3+, Ni2+, or Co2+, with or without Z-WEHD-FMK: (A, C and E) Percentages of dead cells; (B, D and F) Total numbers of cells (viable and dead). Cells were incubated under cell culture conditions with the indicated concentrations of ions in the presence or absence of Z-WEHD-FMK (20 μM), a caspase-1 inhibitor, for 18 to 24h following a 6h priming incubation with lipopolysaccharide (LPS; 500 ng/mL). Cells were counted by hemocytometry and dead cells were identified using the trypan blue dye-exclusion method. The total numbers of cells (viable and dead) were expressed as a ratio of the total number of cells in the negative control (cells with no ions and no Z-WEHD-FMK). An asterisk (*) indicates a significant difference (p<0.05) between a given ion concentration with or without Z-WEHD-FMK and the negative control. A dagger (†) indicates a significant difference (p<0.05) between the results obtained with and without Z-WEHD-FMK at a given ion concentration. Data are presented as means ± SEM of 3–4 independent experiments performed in triplicate. (TIF) [file pone.0199936.s001.tif]

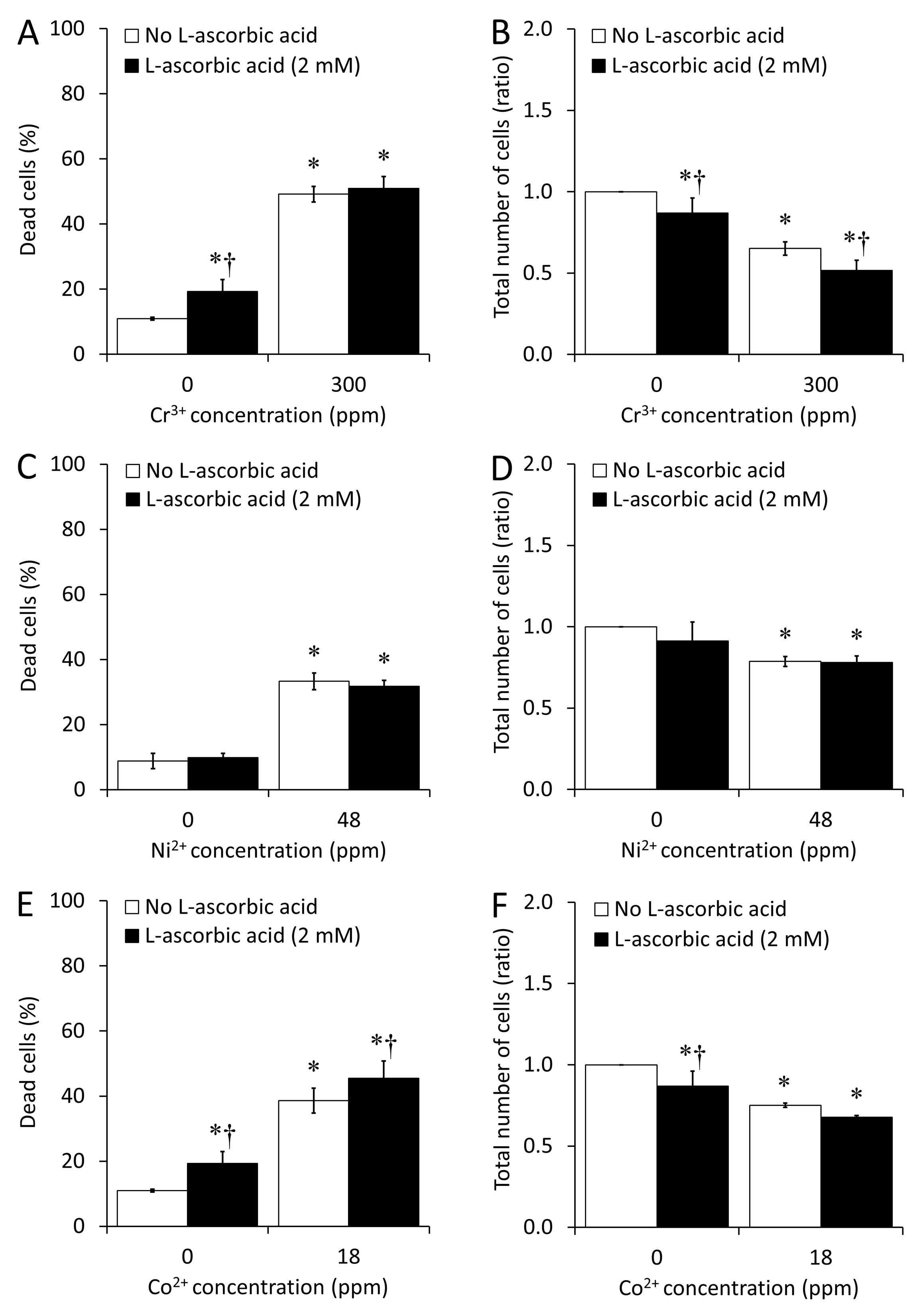

Supplement: S2 Fig — Mortality of bone marrow-derived macrophages (BMDM) after exposure to Cr3+, Ni2+, or Co2+, with or without L-AA: (A, C and E) Percentages of dead cells; (B, D and F) Total numbers of cells (viable and dead). Cells were incubated under cell culture conditions with the indicated concentrations of ions in the presence or absence of L-AA (2 mM), an antioxidant, for 18 to 24h following a 6h priming incubation with lipopolysaccharide (LPS; 500 ng/mL). Cells were counted by hemocytometry and dead cells were identified using the trypan blue dye-exclusion method. The total numbers of cells (viable and dead) were expressed as a ratio of the total number of cells in the negative control (cells with no ions and no L-AA). An asterisk (*) indicates a significant difference (p<0.05) between a given ion concentration with or without L-AA and the negative control. A dagger (†) indicates a significant difference (p<0.05) between the results obtained with and without L-AA at a given ion concentration. Data are presented as means ± SEM of 3 independent experiments performed in triplicate. (TIF) [file pone.0199936.s002.tif]

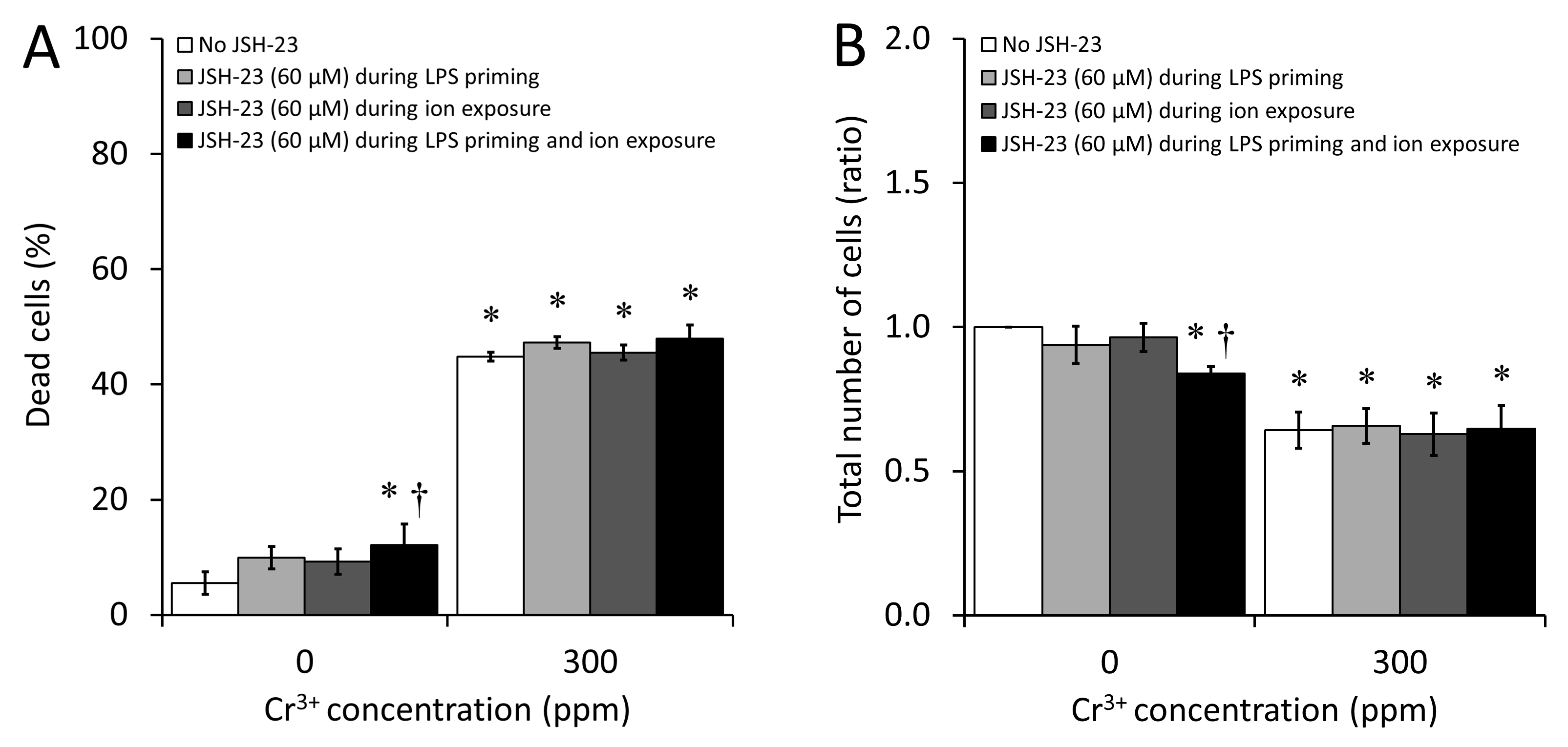

Supplement: S3 Fig — Mortality of bone marrow-derived macrophages (BMDM) after exposure to Cr3+ with or without JSH-23: (A) Percentages of dead cells; (B) Total numbers of cells (viable and dead). Cells were incubated under cell culture conditions with or without Cr3+ (300 ppm) in the presence or absence of JSH-23 (60 μM), an NF-κB inhibitor, for 18 to 24h following a 6h priming incubation with 500 ng/mL of lipopolysaccharide (LPS). In total, four JSH-23 conditions were tested: no JSH-23 (white columns), JSH-23 present exclusively during the priming incubation with LPS (light gray columns), JSH-23 present exclusively during the activation incubation with Cr3+ (dark gray columns), and JSH-23 present during both incubations (black columns). Cells were counted by hemocytometry and dead cells were identified using the trypan blue dye-exclusion method. The total numbers of cells (viable and dead) were expressed as a ratio of the total number of cells in the negative control (cells with no ions and no JSH-23). An asterisk (*) indicates a significant difference (p<0.05) between a given ion concentration with or without JSH-23 and the negative control (cells with no ions and no JSH-23). A dagger (†) indicates a significant difference (p<0.05) between the results obtained with and without JSH 23 at a given ion concentration. Data are presented as means ± SEM of 3 independent experiments performed in triplicate. (TIF) [file pone.0199936.s003.tif]
